# Cerebral cysticercosis in a wild Bengal tiger *(Panthera tigris tigris)* in Bhutan: A first report in non-domestic felids^☆^

**DOI:** 10.1016/j.ijppaw.2021.02.003

**Published:** 2021-02-10

**Authors:** Yoenten Phuentshok, Kinley Choden, Cristian A. Alvarez Rojas, Peter Deplazes, Sonam Wangdi, Kuenzang Gyeltshen, Karma Rinzin, Nirmal Kumar Thapa, Tenzinla Tenzinla, Dechen Dorjee, Marc Valitutto, Martin Gilbert, Boripat Siriaroonrat, Waleemas Jairak, Chutchai Piewbang, Puspa Maya Sharma, Tshewang Dema, Ratna Bahadur Gurung

**Affiliations:** a Food and Agriculture Organization of the United Nations, Bangkok, 10200, Thailand; b Nature Conservation Division, Department of Forests and Park Services, Ministry of Agriculture and Forests, Taba, Bhutan; c Institute of Parasitology, Vetsuisse Faculty, University of Zurich, Zurich, Switzerland; d Animal Health Division, Department of Livestock, Ministry of Agriculture and Forests, Thimphu, Bhutan; e National Centre for Animal Health, Department of Livestock, Ministry of Agriculture and Forests, Serbithang, Bhutan; f World Wildlife Fund, Washington DC, 20037, USA; g EcoHeatlh Alliance, New York, NY, 10018, USA; h Cornell Wildlife Health Center, College of Veterinary Medicine, Cornell University, Ithaca, NY, 14853, USA; i Bureau of Conservation and Research, Zoological Park Organization, Bangkok, 10800, Thailand; j Faculty of Veterinary Science, Chulalongkorn University, Bangkok, 10330, Thailand

**Keywords:** *Taenia solium*, Bengal tiger, *Panthera tigris tigris*, Bhutan, Conservation medicine, Neurocysticercosis, One health, First report

## Abstract

The endangered Bengal tiger (*Panthera tigris tigris*) is a keystone species playing an essential role in ecology as well as in the social and spiritual lives of the Himalayan people. The latest estimate of the Bengal tiger population in Bhutan accounts for 103 individuals. Infectious organisms, including zoonotic parasites causing high burden in human health, have received little attention as a cause of mortality in tigers. Taeniosis/cysticercosis, caused by the cestode *Taenia solium*, is considered one of the major neglected tropical diseases in Southeast Asia. We present here a case of neurocysticercosis in a Bengal tiger showing advanced neurological disease outside Thimphu, the capital city of Bhutan. After palliative care, the animal died, and necropsy revealed multiple small cysts in the brain. Here we show the presence of two genetic variants of *T*. *solium* in the parasite material collected based on PCR and sequencing of the complete *cox1* and *cytB* genes. The sequences form a discrete branch within the Asia plus Madagascar cluster of the parasite. On other hand, tests for feline morbillivirus, feline calicivirus, canine distemper virus, Nipah, rabies, Japanese encephalitis, feline leukaemia and feline immunodeficiency virus were negative. In contrast, PCR for feline herpesvirus was positive and a latex agglutination test revealed an elevated antibody titer against *Toxoplasma gondii* (titer 1:256). The molecular examination of taeniid eggs isolated from the tiger faeces produced sequences for which the highest homology in GenBank is between 92% and 94% with *T. regis and T. hydatigena*. This fatal case of *T. solium* neurocysticercosis, a disease previously unrecorded in tigers or other non-domestic felids, demonstrates an anthropogenically driven transmission of a deadly pathogen which could become a serious threat to the tiger population.

## Highlights

• This is the first report of a fatal infection with *Taenia solium* in a Bengal tiger.
• There is a need for research into infectious disease threats to tigers in Bhutan.
• The importance of a ‘One Health’ approach is demonstrated and recommended.
• Non-domestic felids.

## Graphical abstract

**Figure:**
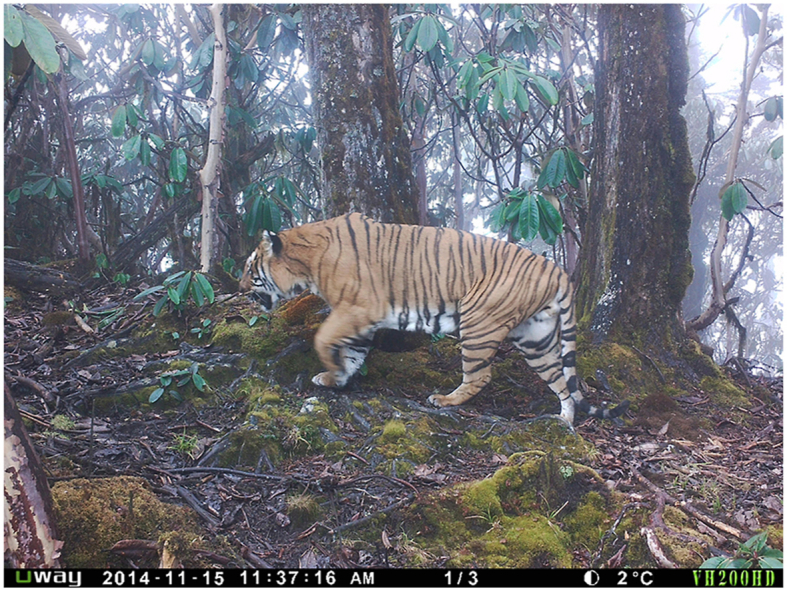

## 1 Introduction

The International Union for Conservation of Nature (IUCN) has listed tigers as endangered since 1969 with the global tiger numbers and range continue to decline. By 2010, fewer than 3500 animals occupied approximately 7% of their historical global range (Sanderson et al., 2010; Seidensticker, 2010; Walston et al., 2010). With more than 70% of its land covered by forest, Bhutan is a hotspot for wild felids (Tempa et al., 2013). The Bengal tiger (*Panthera tigris tigris*, Linnaeus 1758) is a keystone species playing an important role in ecology as well as the social and spiritual lives of the Bhutanese people. An estimated population of 103 adult tigers roam undisturbed in Bhutan (Ministry of Agriculture and Forests, 2015) with their habitat ranging from the southern foothills bordering India at 150 m above sea level (masl) to alpine forests and the high Himalayas adjoining the Tibetan Autonomous Region to the north at 4300 masl (Jigme and Tharchen, 2012). The tiger population in Bhutan is thriving compared to many other regions, but poaching is still considered the main threat to their conservation. By contrast, little attention has been given to the impact that zoonotic pathogens and disease might represent to tiger conservation in Bhutan. This situation warrants re-evaluation following the case of neurocysticercosis reported in this article.

Cysticercosis due to infection with the cestode *Taenia solium* is a widely distributed zoonosis with the highest transmission in South America, India, Africa and Southeast Asia but is probably under-recognized in many other endemic countries (Flisser et al., 2011; Ito et al., 2019). Bhutan is considered part of an endemic area of infection with northern India and Nepal (Ito et al., 2019). In Bhutan, there has recently been an increasing number of epilepsy cases among human patients attributed to neurocysticercosis (Brizzi et al., 2016; Bruno et al., 2017; Health, 2016). The life cycle of the tapeworm *T. solium* takes place in two hosts, with humans acting as the definitive host and domestic pigs as the intermediate host. Besides humans, intestinal infection has been established experimentally in other primates including lar gibbons *(Hylobates lar)* and chacma baboons (*Papio ursinus*), as well as in immunosuppressed laboratory animals (Flisser et al., 2011). However, the potential for a sylvatic cycle of *T. solium* involving wild primates and intermediate hosts such as wild boars (*Sus scrofa*) has yet to be documented. Humans acquire intestinal infections of *T. solium* through ingestion of cysticerci in undercooked infected pork. The cysticerci develop within 9–10 weeks into adult gravid tapeworms which can persist for several months as subclinical patent infections (Flisser et al., 2011). During the patency, eggs are excreted in proglottids or free in the stool of the infected humans. Pigs ingest embryonated eggs from contaminated soil, water, food or through coprophagia. The eggs hatch in the pig's intestine and the activated oncospheres penetrate the mucosa, migrate into the bloodstream and settle in tissues where they form the larval stages containing an invaginated scolex. In humans exposed to *T. solium* eggs, the oncospheres invade the mucosa following antiperistalsis (patients with intestinal infections), by hand-mouth transmission after contact with egg contaminated surfaces or by ingestion of contaminated food, water or soil. The migrating oncospheres encyst in various organs leading to clinical signs associated with their location. Cysticerci developing in the central nervous system lead to neurological symptoms (neurocysticercosis), causing epileptic seizures and increased intracranial pressure, which can be fatal (Garcia and Del Brutto, 2005; García et al., 2010).

## 2 Materials and methods

### 2.1 Clinical presentation and necropsy

**Fig. 1:**
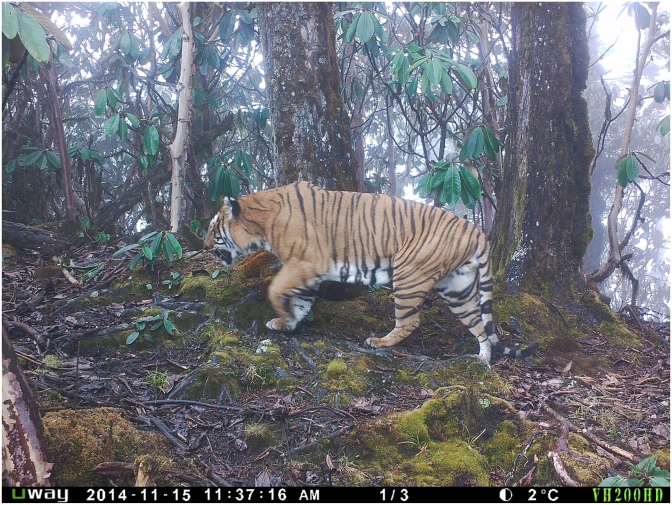
Camera trap image of the tiger infected with *Taenia solium* reported in this article captured on the 15^th^ of November 2014. The image was taken during a survey of the Bengal tiger population at national level in Bhutan. Copyright: Nature Conservation Division, DoFPS, MoAF, Bhutan.

On March 21, 2018, an adult male tiger, subsequently identified using old remote camera trap images as a known individual of 9 years of age (Fig. 1) emerged from the forest close to the capital city of Thimphu (at 2300 masl and home to approximately 115,000 inhabitants). Residents of Chamjekha, a residential complex in Kabesa suburb located about 6 km north of Thimphu city shared photos and videos of the tiger on social media, which subjectively showed the animal being indifferent to its surroundings. The Nature Conservation Division (NCD), Department of Forests and Park Services (DoFPS) of the Ministry of Agriculture and Forests (MoAF) intervened and captured the tiger from Taba (89°38′45″ E; 27°30′49″ N), another suburb adjacent to Kabesa in the early hours of March 22, 2018, using 450 mg of tiletamine and zolazepam (Zoletil®100, Virbac, Pty. Australia); the dose (4 mg/kg body weight) was based on an estimated body weight of 115 kg. The tiger exhibited clinical signs consistent with a compromised nervous system including uncoordinated gait, stumbling, walking in clockwise circles with head held down, head pressing, lack of aggression and indifference to surrounding objects and people. A small bruised wound on the forehead skin was evident of constant pressing of the head on fixed objects. The tiger was cared for in the NCD Wildlife Rescue and Rehabilitation Centre for fifteen days. Initially, he was treated orally with antibiotics (Cefotaxime) and vitamin B. The tiger was active and eating (~10 kg beef) and drinking (~4 L water) daily but the health conditions kept deteriorating daily. On the 6th day, the animal became inactive, developed sunken eyes and hyporexia, consuming only small volumes of water but no meat. Despite supportive care, on the 12th day, the tiger's neurological condition significantly advanced, as exhibited by an inability to stand (but in sternal recumbency with the head elevated), staring, with no response to visual or auditory stimuli and an inability to hold food in its mouth. By the 13th day, the tiger's condition progressed to lateral recumbency with no response to touching or painful stimuli. The animal died on the 6^th^ of April 2018; necropsy was performed within 5 hours of death.

The necropsy was performed following established protocols, and samples of different organs were collected and fixed in 10% formalin and/or directly frozen. Oral, rectal and conjunctival swabs and faeces were also collected. The cysts recovered on necropsy were triturated and frozen at −20 °C and the material was sent to the Institute of Parasitology, University of Zurich together with taeniid eggs isolated from faeces.

### 2.2 Histopathology

Formalin-fixed tissues from liver, lung, spleen, kidney, diaphragm, testicle, pancreas, trachea, heart and hind pad were shipped to the College of Veterinary Medicine, Cornell University for histological examination.

### 2.3 Diagnosis of viral and parasitic infections in serum and tissues

Ancillary diagnostics were performed in Thailand at the National Institute of Animal Health, Mahidol University, Chulalongkorn University, and in the United States at Cornell University to determine the presence or exposure to a variety of viral and apicomplexan pathogens. Details of the methods used are provided in Table 1.

**Table 1:** Summary of virology and Toxoplasma results from analyses performed at Mahidol University, Chulalongkorn University, Cornell University, and the National Institute of Animal Health (NIAH). Tests performed included reverse transcription-polymerase chain reaction (RT-PCR), polymerase chain reaction (PCR), enzyme-linked immunosorbent assay (ELISA), hemagglutinin inhibition (HI), latex agglutination test (LAT), virus neutralisation test (VNT) and Western blot (WB).

Pathogen	Method	Specimen tested	Lab	Result
Molecular analysis:				
Canine distemper virus	RT-PCR	Rectal swab, whole blood	Mahidol	Negative
Canine distemper virus	RT-PCR	Brain	Cornell	Negative
Nipah virus	RT-PCR	Nasal and oral swab	Mahidol	Negative
Rabies virus	RT-PCR	Oral swab	Mahidol	Negative
Japanese encephalitis virus	RT-PCR	Blood	Mahidol	Negative
Feline morbillivirus	RT-PCR	Rectal swab and blood	Chulalongkorn	Negative
Panparamyxovirus	RT-PCR	Nasal and rectal swab	Chulalongkorn	Negative
Feline calicivirus	RT-PCR	Nasal and conjunctival swab	Chulalongkorn	Negative
Feline herpesvirus	PCR	Oral and rectal swab	Chulalongkorn	Positive
Feline leukaemia virus	ELISA	Serum	Cornell	Negative
Serological analyses:				
Japanese encephalitis virus	HI	Serum	Mahidol	Negative
Toxoplasma gondii	LAT	Serum	NIAH	Positive (1:256)
Canine distemper virus	VNT	Serum	Chulalongkorn	Negative
Feline immunodeficiency virus	WB	Serum	Cornell	Negative

### 2.4 Molecular analysis of the brain lesion

An aliquot of the sample was washed three times in PBS(physiological phosphate buffered saline), and total DNA was extracted using the QIAGEN DNeasy Blood & Tissue Kit. DNA was used as a template for a multiplex PCR as previously described (Trachsel et al., 2007). PCR products were visualised in agarose gel 2%, purified with the MinElute QIAGEN kit and sequenced at Microsynth (Switzerland) using the PCR primers. Additional PCRs were performed to amplify the full length of the *cox1* (cytochrome c oxidase I) and *cytb* (cytochrome b) genes of the parasite for phylogenetic analysis according to Nakao et al. (2002). PCR products were cloned using the TOPO TA Cloning Kit (Thermo Fisher Scientific), five clones were sequenced per gene using vector primers. Sequences of the *cox1* and *cytB* genes and all similar sequences of the same length from *T*. *solium* were collected from GenBank and aligned using the software Geneious R10 (https://www.geneious.com). Phylogenetic trees were inferred for each gene with the neighbour-joining method using *Echinococcus multilocularis* as an outgroup.

### 2.5 Microscopic and molecular analysis of faeces

At the National Centre for Animal Health, Bhutan, faecal samples from the tiger were processed following the floatation and sieving method as described by Mathis et al. (1996). The sediment of the 21 μm filter was examined microscopically and fixed in ethanol 70% and sent to the Institute of Parasitology (Zurich, Switzerland) for subsequent molecular analysis. The sediment was subjected to alkaline lysis to isolate DNA as previously described (Štefanić et al., 2004). The DNA was used as a template for a multiplex PCR (Trachsel et al., 2007) to get an initial identification of the taeniid eggs. Additional PCR was performed amplifying a section of the *nadh* gene (NADH dehydrogenase subunit 1) (Bowles and McManus, 1993). PCR products were sequenced and compared with the NCBI database. Cladograms were inferred with the neighbour-joining method using the same gene section from the reference mitochondrial genome of other *Taenia* species.

## 3 Results

### 3.1 Necropsy

**Fig. 2:**
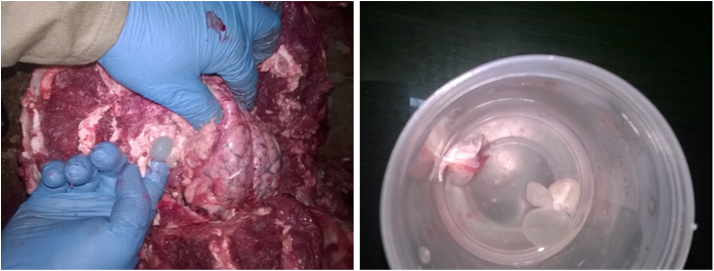
Cysts identified at necropsy of the Bengal tiger in Bhutan (left) and isolated in a jar (right).

Healthy and intact coat was observed except a lacerated wound in the middle of the forehead; the injury could have occurred from pressing its head against the cage. A mark of its own (lower) canine teeth at either side of the upper lips were observed; the right lower canine was missing and the left incisor was broken at the root. The liver was congestive, haemorrhagic and friable while extensive haemorrhages were noted in the internal mucosa of the small intestine. Both lobes of the lungs were collapsed and ecchymotic. The mesenteric lymph nodes were slightly enlarged and haemorrhagic. Both kidneys appeared slightly enlarged, and haemorrhages were noted in the cortex and medulla. The brain contained two clear fluid-filled cysts approximately 1–2 cm diameter buried deep inside the brain tissue. The larger cyst was located on the left lobe of the cerebrum while the slightly smaller cyst was lodged in the right brain (Fig. 2).

### 3.2 Molecular analysis of the cyst

**Fig. 3:**
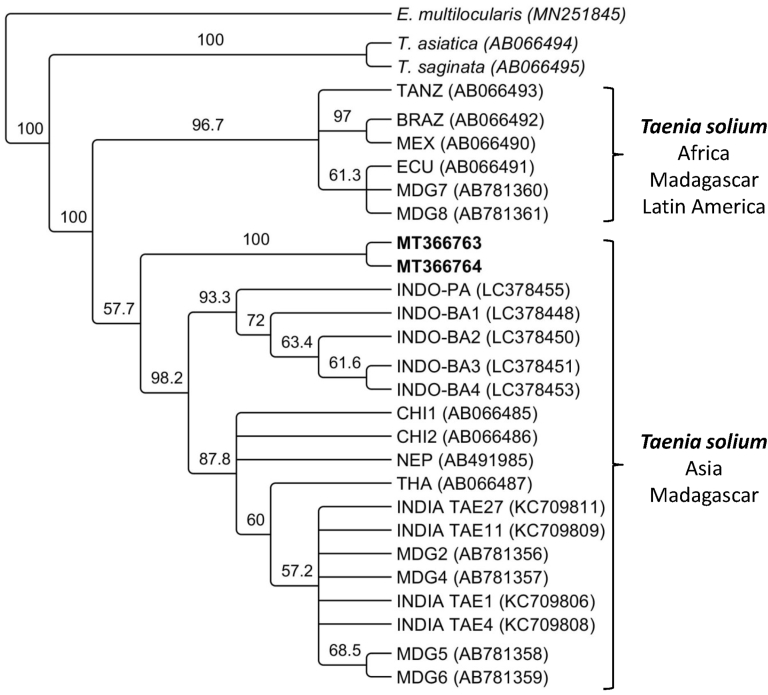
Cladogram showing the relation between the sequences of the *cox1* gene of *Taenia solium* from the present study highlighted in bold (MT366763-MT366764) and unique published sequences of the *cox1* gene available in GenBank from Tanzania (TANZ), Brazil (BRA), Mexico (MEX), Ecuador (ECU), Madagascar (MDG), Indonesia (INDO-PA, INDO-BA), China (CHI), Nepal (NEP), Thailand (THA) and India (TAE). Sequences for the *cox1* gene from *Taenia saginata* (AB066495) and *T*. *asiatica* (AB066494) were also included. The *cox1* sequence of *Echinococcus multilocularis* (MN251845) was used as an outgroup.

**Fig. 4:**
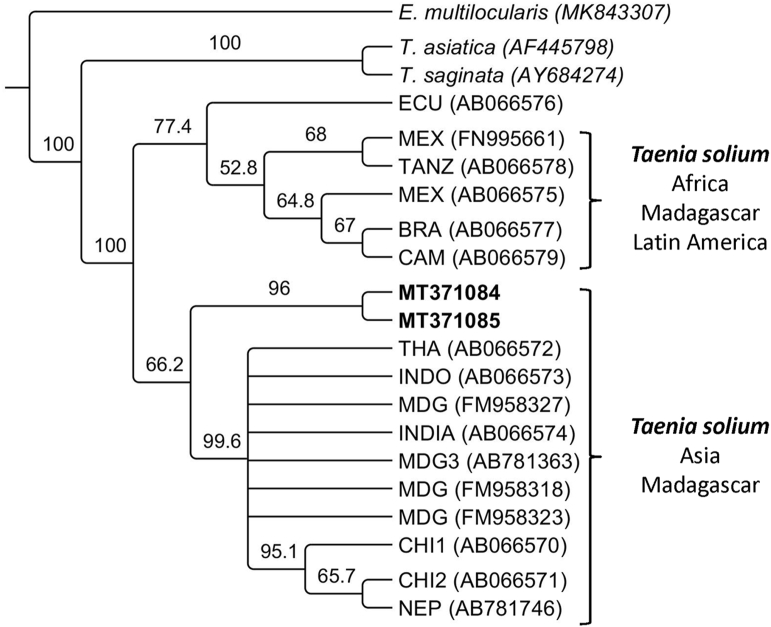
Cladogram showing the relation between the sequences of the *cytB* gene of *Taenia solium* from the present study highlighted in bold (MT371084-MT371085) and unique published sequences of the *cytB* gene available in GenBank from Ecuador (ECU), Tanzania (TANZ), Mexico (MEX), Brazil (BRA), Cameroun (CAM), Thailand (THA), Indonesia (INDO), Madagascar (MDG), INDIA, China (CHI) and Nepal (NEP). The sequences for the *cytB* gene from *Taenia saginata* (AB066581) and *Taenia asiatica* (AB066580) are also included. The *cytB* sequence from *Echinococcus multilocularis* (MK843307) was used as an outgroup.

The PCR, according to Trachsel et al. (2007) produced a single band (267bp) which sequence showed 99.5% homology with the corresponding section of the *T. solium* full mitochondrial genome (AB086256). The PCR products of the *cox1* (1,620bp) and the *cytB* (1,068bp) were cloned and two different variants were identified per gene differing only in two nucleotides between them. Sequences were deposited in GenBank Accession numbers: MT366763- MT366764 for *cox1* and MT371084-MT371085 for *cytB*. The cladograms showing the relationship between the sequences for *cox1* and *cytB* from different isolates of *T*. *solium* deposited in GenBank is shown in Fig. 3, Fig. 4 respectively. The sequences of both genes cluster together with the Asia + Madagascar group of *T. solium* previously described by Nakao et al. (2002).

### 3.3 Histopathology

The most striking finding was congestion of the liver (severe) and lung (mild). The liver congestion appeared diffuse (rather than targeted to a specific region), with erythrocytes expanding the sinusoids leading to disarray and fragmentation of hepatic chords without signs of cellular necrosis. No significant lesions were identified in the other tissues.

### 3.4 Diagnosis of viral and parasitic infections in serum and tissues

The results of molecular and serological analyses are summarized in Table 1. All tests were negative except for a PCR for feline herpesvirus (FHV); and the latex agglutination test for antibodies against *Toxoplasma gondii* (titer 1:256).

### 3.5 Molecular analysis of taeniid eggs in faeces

**Fig. 5:**
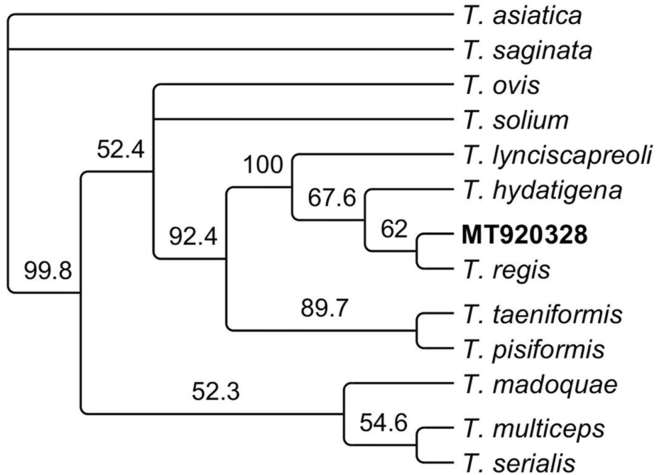
Cladogram showing the relation between the sequence of the *nad1* gene (491bp) amplified from the sediment of the 21 μm filter of the sieving method for isolation of taeniid eggs from faeces of the tiger analysed in this study highlighted in bold (MT920328); together with the sequence of the same gene of different *Taenia* spp.

Faecal examination revealed the presence of taeniid eggs. A band of 267bp was amplified using a multiplex PCR to discriminate between *Echinococcus* and other cestodes including *Taenia*. The closest homology in GenBank for this sequence was around 94% with the corresponding section of the complete genome of *T*. *regis* (AB905198) and *T*. *hydatigena* (GQ228819). Subsequently, a section of the *nadh* gene (491bp) was amplified and its sequence (accession number: MT920328) showed a homology around 92% with the corresponding section of the genome of *T*. *hydatigena* (FJ518620) and *T*. *regis* (AB905198). The proximity of this sequence with *T*. *regis* is evident in the cladogram in Fig. 5 which includes other *Taenia* species.

## 4 Discussion

Neurological disease in wild tigers has recently gained prominence following the recognition that Canine Distemper Virus (CDV) represents a threat to the conservation of Amur tigers (*P. tigris altaica*) in the Russian Far East (Gilbert et al., 2015; Seimon et al., 2013). The lack of any serological or molecular evidence for active or resolved CDV infection in the tiger suggested an alternative aetiology for this case. The identification of *T. solium* in two cerebral cysts and clinical signs consistent with intracranial lesion suggested that neurocysticercosis was the only supported diagnosis in this case. There have been very few published reports of infectious disease in free-ranging tigers (Gilbert et al., 2015) and our finding highlight the importance of considering alternative aetiologies to CDV when wild tigers present with neurological signs.

The initial molecular analyses performed here confirmed that *T. solium* was responsible for the cerebral cysts present in the tiger. Additionally, two variants of each gene (*cox1* and *cytB*) were identified in this study, since there were two cysticerci in the brain of the tiger (which were pooled before molecular analysis). Each of them was likely caused by different variants of the parasite. Fig. 3, Fig. 4 show that sequences identified in the present study are part of the Asia and Madagascar group of *T*. *solium*, which is distinct from the group of isolates from Africa, Latin America and Madagascar. However, they form a separate branch within the Asian and Madagascar group. So far, this group comprises samples from China, India, Indonesia, Taiwan and Thailand and Madagascar. In the case of Madagascar, it is well known that *T. solium* of African-American and Asian genotypes are present due to animal trade from both regions towards the island (Michelet et al., 2010). In the case of Bhutan, its geopolitical isolation has likely contributed to the establishment of distinct variants of *T*. *solium* which have not been previously described. Further examination of *T*. *solium* isolates from humans, pigs or wild boar are needed to fully understand the molecular epidemiology of *T*. *solium* in Bhutan and neighbouring countries.

To our knowledge, this is the first case of neurocysticercosis recorded in a wild or captive tiger. Cerebral cysticercosis, also known as neurocysticercosis, caused by the larval stage (metacestode) cysticercus cellulosae of *T. solium* (Cyclophyllida: Taeniidae), has been identified as a major public health problem in low and middle-income countries (Donadeu et al., 2016). Besides its normal intermediate host, the domestic pig, the metacestode of *T. solium* has been reported from a wide range of terrestrial (Abuladze and Mifʻal tirgume ha-madaʻ, 1970), as well as from a few marine mammals (De Graaf et al., 1980). However, although these occasional infections are of clinical importance to the infected individuals, they are thought to represent “dead-end hosts” and are epidemiologically unimportant at a population level. Apart from human, cases of cerebral *T. solium* cysticercosis have been reported in domestic dogs (Rogers et al., 1989; Rommel et al., 2000) and to a lesser extent in domestic cats (Carillo Melgar and Quiroz Romero, 1974; Mazzotti et al., 1965). Interestingly, a case of *T. solium* neurocysticercosis was found in a cat in South Africa (Schwan et al., 2002). Clinical symptoms were observed around 6 h after deworming with praziquantel, a drug which can increase the pathogenicity of the infection by enhancing local inflammation around the dead parasite (Schwan et al., 2002). Another rare case of a cerebral *Taenia crassiceps* cysticercosis in a domestic cat was described in the USA (Wünschmann et al., 2003), a *Taenia* sp. probably also endemic for Bhutan. Rare cases of coenurosis in cats caused by *T. serialis* have been reported (Hayes and Creighton, 1978; Huss et al., 1994; Orioles et al., 2014; Slocombe et al., 1989; Smith et al., 1988). In Bhutan, *T. multiceps* is an important species causing coenurosis with high mortalities in young yaks (Dorji, 2002). However, there have been no cases of *T. multiceps* in both domestic and wild felids in Bhutan.

Domestic and wild cats are the definitive hosts of the apicomplexan parasite *Toxoplasma gondii*, but clinical disease is rare in most feline species (Elmore et al., 2010). Antibodies to *T. gondii* were detected in 62% (95% CI: 45.7–76.0%, n = 42) of wild Amur tigers sampled in the Russian Far East (Goodrich et al., 2012) and so their detection in the Bhutan tiger was not unexpected. Although clinical toxoplasmosis has been reported in a captive Amur tiger with profuse diarrhoea and wasting (Dorny and Fransen, 1989), in the case presented, *Toxoplasma* seropositivity alone is not indicative for the clinical presentation of the animal. FHV causes an upper respiratory and ocular infection in cats including tigers (Sun et al., 2014). Extended infections due to latency increase the likelihood of detection, and the absence of neurological disease associated with FHV infections suggest the detection of the virus was also incidental. Immunosuppression has contributed to other cysticercoses in atypical dead-end hosts as described for *Versteria sp.* or *T. crassiceps* cysticercoses in primates including humans (Deplazes et al., 2019). However, in this case, other pathogens associated with immunosuppression including CDV, feline immunodeficiency virus and feline leukaemia were not detected.

Interestingly, the molecular analysis of taeniid eggs isolated from faeces reveal sequences which share between 92 and 94% similarity with *T*. *regis* and *T*. *hydatigena*. The electropherograms of these sequences showed clear single peaks and good quality which allow us to be confident that these PCR products are not artefacts or the consequence of a mixed infection with different *Taenia* species. Phylogenetic analysis showed that it is likely that the *Taenia* eggs found in the tiger faeces could belong to a non previously described species (Fig. 5). However, this could only be confirmed if a morphological and molecular analysis of the adult worm could be carried out. Unfortunately, it was not possible to have access to such material. The sequence of the *nad1* gene from this study is also different from sequences from *T*. *regis* and *T*. *hydatigena* isolated from carnivores and felids in Kenya (Zhang et al., 2007). Little is known about *Taenia* species infecting tigers in Asia. In the case of Africa, *T*. *simbae*, a rare species in lions, was characterized in the past based on morphology and was described to be similar to *T*. *regis* (Dinnik and Sachs, 1972). To date, there is no molecular data for *T*. *simbae* and also there is no record of its presence in Asia. Future characterisation of taeniids from tigers could clarify whether the *Taenia* species infecting the tiger intestine is related to *T*. *simbae* or is a different species of *Taenia*.

The mechanism for *T. solium* exposure in this unusual case of neurocysticercosis in a wild Bengal tiger is unknown. It is possible that the tiger may have ingested infective *T. solium* eggs through direct consumption of human faeces or consumption of water or food contaminated with human faeces. Camera trapping records of the Nature Conservation Division shows that the home range of this tiger extended to the outskirts of Thimphu, which is the most populated city in Bhutan with a population of around 115,000 inhabitants. Bhutan is known to be an endemic area of *T. solium* neurocysticercosis with cases throughout the country including urban populations (Brizzi et al., 2016). Open defecation in forests or bushes is a common practice. With an increasing number of trekkers and commuters passing through the tiger's habitat, this environmental contamination is a plausible mechanism for exposure. Considering the escalating pressures on the tiger habitat and their increasing proximity to people, it is unknown whether the Bhutanese case was an unusual and incidental finding or an indication of a broader problem. With global tiger populations now below 1000 breeding females (Sanderson et al., 2010; Seidensticker, 2010; Walston et al., 2010), this case illustrates the need for better understanding of infectious causes of mortality and its impact on tiger conservation in landscapes that are increasingly dominated by people.

## Funding

Cornell Wildlife Health Centre and Institute of Parasitology, Vetsuisse Faculty, University of Zurich for diagnostic investigations performed. WWF country office, Bhutan for support with camera in the animal enclosure for observation and the Ministry of Agriculture and Forests (MoAF), Royal Government of Bhutan for sample referral.

## Declaration of competing interest

The authors declare that they have no known competing interests that could have appeared to influence the work reported in this paper. All authors approved the final manuscript and its submission.
